# Non-invasive vagus nerve stimulation for acute treatment of high-frequency and chronic migraine: an open-label study

**DOI:** 10.1186/s10194-015-0542-4

**Published:** 2015-06-30

**Authors:** Piero Barbanti, Licia Grazzi, Gabriella Egeo, Anna Maria Padovan, Eric Liebler, Gennaro Bussone

**Affiliations:** Headache and Pain Unit, Department of Neurological Motor and Sensorial Sciences, IRCCS San Raffaele Pisana, Via della Pisana 235, 00163 Rome, Italy; Headache Center, Carlo Besta Neurological Institute and Foundation, Via Celoria 11, 20133 Milan, Italy; Kiara Association, Via Olivia 4. Giaveno (TO), 10094 Turin, Italy; ElectroCore LLC, Basking Ridge, 150 Allen Road, Suite 201, Basking Ridge, NJ 07920 USA

**Keywords:** Migraine, Neuromodulation, Vagus nerve, Acute treatment, Patient preference, Disability

## Abstract

**Background:**

The treatment of migraine headache is challenging given the lack of a standardized approach to care, unsatisfactory response rates, and medication overuse. Neuromodulation therapy has gained interest as an alternative to pharmacologic therapy for primary headache disorders. This study investigated the effects of non-invasive vagus nerve stimulation (nVNS) in patients with high-frequency episodic migraine (HFEM) and chronic migraine (CM).

**Findings:**

In this open-label, single-arm, multicenter study, patients with HFEM or CM self-treated up to 3 consecutive mild or moderate migraine attacks that occurred during a 2-week period by delivering two 120-s doses of nVNS at 3-min intervals to the right cervical branch of the vagus nerve. Of the 50 migraineurs enrolled (CM/HFEM: 36/14), 48 treated 131 attacks. The proportion of patients reporting *pain relief*, defined as a ≥50 % reduction in visual analog scale (VAS) score, was 56.3 % at 1 h and 64.6 % at 2 h. Of these patients, 35.4 % and 39.6 % achieved *pain*-*free status* (VAS = 0) at 1 and 2 h, respectively. When all attacks (*N* = 131) were considered, the pain-relief rate was 38.2 % at 1 h and 51.1 % at 2 h, whereas the pain-free rate was 17.6 % at 1 h and 22.9 % at 2 h. Treatment with nVNS was safe and well tolerated.

**Conclusion:**

Non-invasive vagus nerve stimulation may be effective as acute treatment for HFEM or CM and may help to reduce medication overuse and medication-associated adverse events.

## Findings

### Introduction

Migraine, a highly disabling neurological disorder, is characterized by recurrent moderate to severe attacks associated with vegetative symptoms [1]. Patients with frequent attacks may overuse medications, leading to migraine chronification and medication-overuse headache. During the last decade, neuromodulatory approaches have been developed for the management of headaches that do not respond adequately to therapy [2]. Invasive neurostimulation targeting the hypothalamus, sphenopalatine ganglia, and occipital, supraorbital, or auriculotemporal nerves has yielded encouraging results [2]. Vagus nerve stimulation (VNS), an invasive procedure, is approved for medically refractory epilepsy and depression [3, 4] and has demonstrated clinical benefit in intractable migraine with comorbid depression [5]. Experimentally, VNS has modulated neurotransmitters, influenced cerebral metabolism [6] and blood flow [7] in the limbic system and pain matrix regions, and exerted antinociceptive effects in acute and inflammatory pain models [8, 9]. Proposed mechanisms of VNS in pain pathways may involve modulation of excess glutamate levels in the trigeminal nucleus caudalis, effects on pain control centers, and modulation of cortical excitability [9–11].

A non-invasive VNS device (nVNS; gammaCore®) has been developed and is CE-marked for acute and prophylactic treatment of primary headache disorders including migraine and cluster headache [12]. In a recent open-label study of 30 episodic migraineurs, nVNS was effective in the acute treatment of migraine attacks and resulted in a 2-h pain-free rate of 22 % [11]. To further examine the clinical benefit of nVNS reported in the aforementioned study, we evaluated the acute effects of nVNS on migraine attacks at 1 and 2 h in a larger patient population with high-frequency episodic migraine (HFEM; ≥8 headache days per month, with or without aura) or chronic migraine (CM; ≥15 headache days per month) [1, 13].

## Methods

In this open-label, single-arm, multicenter study, 50 patients aged 18 to 65 years who were experiencing HFEM or CM [1, 13] were consecutively enrolled between February 1, 2013, and October 1, 2013, at the Headache and Pain Unit of the IRCCS San Raffaele Pisana in Rome, Italy, and the Headache Center of the Carlo Besta Neurological Institute and Foundation in Milan. The study protocol was approved by the **San Raffaele Pisana** institutional review board **(10/2013)**, and all patients who were enrolled in the study provided written informed consent. The study population excluded patients with a history of cerebrovascular, cardiovascular, or atherosclerotic disease (including carotid artery disease, heart arrhythmias, or syncope) or any significant neurological or systemic disorder and patients with an implanted electrical device.

At monthly educational meetings involving groups of 3 to 6 patients as well as neurologists and counselors, patients were instructed on how to use the nVNS device and were invited to describe their experiences with migraine and how they usually managed migraine attacks. Patients received basic information on vagus nerve physiology and vagal neurostimulation and watched a video demonstrating how nVNS is believed to work. Prior to study initiation, patients were actively encouraged to use nVNS and received training on the proper use of the device from a physician and via an instructional video.

Patients were instructed to use nVNS to self-treat up to three consecutive migraine attacks that occurred over a 2-week period. For each migraine attack, patients delivered two 120-s doses of electrical stimulation at 3-min intervals to the right cervical branch of the vagus nerve within 20 min of the onset of mild or moderate pain.

Patients were allowed to take a rescue medication if they perceived no reduction in pain 2 h after nVNS treatment. Pain severity was rated using a 0- to 10-cm visual analog scale (VAS) score (0 cm, *no pain*; 1-3 cm, *mild*; 4-6 cm, *moderate*; 7-10 cm, *severe*) at baseline, 1 h, and 2 h. Patients recorded pain severity in a headache diary, along with symptoms of nausea, photophobia, phonophobia, and functional disability (at baseline and 2 h); the use of rescue medications and adverse events were also recorded.

*Pain relief* was defined as a ≥50 % reduction in VAS score. *Pain-free* status was defined as a VAS score of 0. The primary end point was *pain-free* status at 2 h. Secondary end points were *pain relief* at 1 and 2 h; *pain-free* status at 1 h; absence of nausea, photophobia, and phonophobia at 2 h; complete recovery from functional disability at 2 h; use of rescue medication; safety; tolerability; and end-of-study assessment of patients’ satisfaction (5-point scale: 1, *very dissatisfied*, to 5, *very satisfied*) with treatment, their willingness to use the device in the future, and their perceptions regarding the safety of nVNS. Descriptive statistics (ie, mean [standard deviation]) were used to describe categorical data; no other statistical analyses were performed.

## Results

**Fig. 1:**
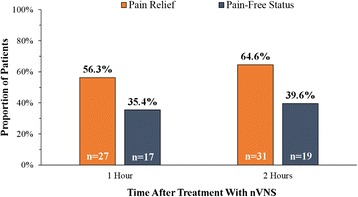
Response to nVNS treatment in 48 Migraineurs. Abbreviations: nVNS, non-invasive vagus nerve stimulation

**Fig. 2:**
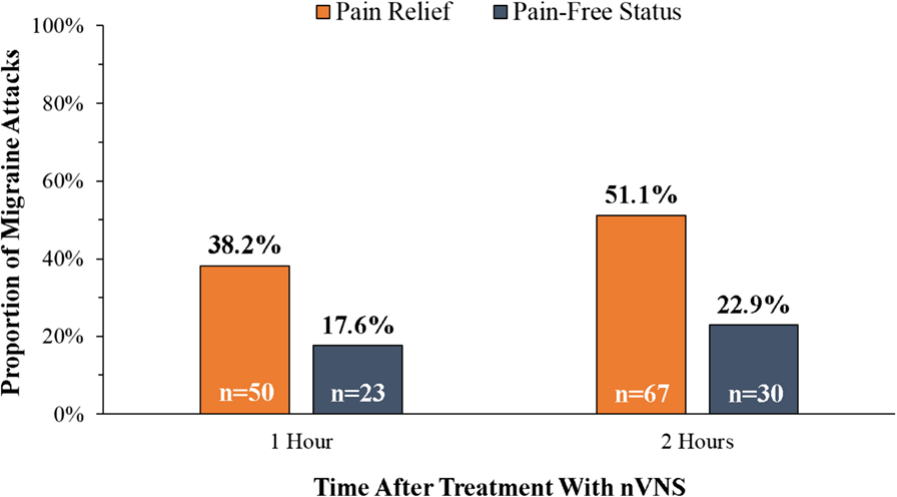
Response to nVNS treatment in 131 Migraine Attacks. Abbreviations: nVNS, non-invasive vagus nerve stimulation

We enrolled 50 patients (female/male: 40/10) affected by CM (*n* = 36) and HFEM (*n* = 14) (Table 1). Two patients with CM did not treat any migraine attacks; the remaining 48 patients treated a total of 131 attacks. Specifically, 30 patients with CM and 6 with HFEM treated 3 attacks each; 4 patients with CM and 7 with HFEM treated 2 attacks each; and 1 patient with HFEM treated 1 attack. After nVNS, 27 of 48 patients (56.3 %) reported *pain relief* at 1 h; of these patients, 35.4 % (n = 17) were pain free. Thirty-one patients (64.6 %) reported *pain relief* at 2 h, of which 39.6 % (n = 19) were pain free (Fig. 1). For all 131 migraine attacks, *pain relie*f was reported for 38.2 % (50 of 131) of attacks at 1 h and for 51.1 % (67 of 131) at 2 h; *pain-free status* was reported for 17.6 % (23 of 131) of attacks at 1 h and for 22.9 % (30 of 131) of attacks at 2 h (Fig. 2). Achievement of *pain-free* status at 1 and 2 h for at least 1 attack was experienced in 33.3 % (11 of 33) of patients treating 3 attacks and 41.7 % (5 of 12) of patients treating 2 attacks (5 of 12).

**Table 1:** Demographic and baseline characteristics of study population

	All	HFEM	CM
	*N* = 50	*n* = 14	*n* = 36
Mean (SD) age, y	43.2 (11.3)	43.2 (12.3)	43.3 (10.8)
Female, n (%)	40 (80)	11 (78.6)	29 (80.5)
Mean (SD) disease duration, y	29.7 (11.2)	30.4 (13.5)	29.5 (10.2)
Mean (SD) number of migraine days per month	15.4 (5.6)	7.9 (2.3)	18.3 (3.3)
Allodynia^a^, n (%)	18 (36)	4 (28.6)	14 (38.9)
Concomitant prophylaxis, n (%)	39 (78)	10 (71.4)	29 (80.6)
Migraine Type, n (%)			
Migraine without aura	14 (28)	14 (100)	0
Medication overuse headache	5 (10)	0	5 (13.9)
Chronic migraine	36 (72)	0	36 (100)
Migraine Pain Location, n (%)			
Unilateral	28 (56)	10 (71.4)	18 (50)
Bilateral	18 (36)	3 (21.4)	15 (41.7)
Unilateral/bilateral	4 (8)	1 (7.2)	3 (8.3)
Duration of Migraine Attacks, n (%)			
≤24 h	17 (34)	5 (35.7)	12 (33.3)
25-48 h	8 (16)	2 (14.3)	6 (16.7)
>48 h	25 (50)	7 (50)	18 (50)

*CM* chronic migraine, *HFEM* high-frequency episodic migraine; *SD* standard deviation

^a^Allodynia was assessed using the Allodynia Symptom Checklist

**Fig. 3:**
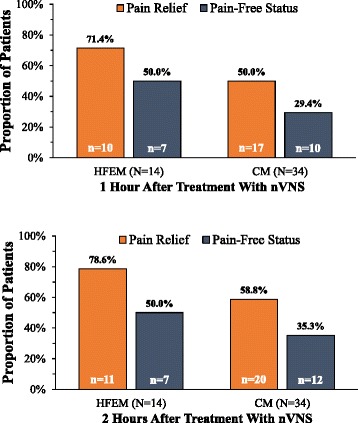
Response to nVNSat 1 and 2 Hours by Patient Type

**Fig. 4:**
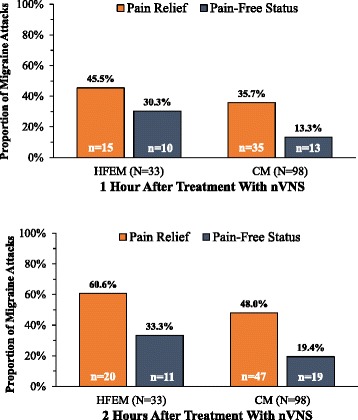
Response to nVNSat 1 and 2 Hours by Migraine Attack Type

**When comparing efficacy of nVNS among patients with CM versus HFEM, we found a consistent trend toward greater efficacy in patients with HFEM. The proportion of patients reporting*pain relief*after nVNS was greater in HFEM at 1 h (HFEM, 71.4 % [10 of 14]; CM, 50.0 % [17 of 34]) and at 2 h (HFEM, 78.6 % [11 of 14]; CM, 58.8 % [20 of 34]); achievement of*pain-free status*was also greater in HFEM at 1 h (HFEM, 50.0 % [7 of 14]; CM, 29.4 % [10 of 34]) and at 2 h (HFEM, 50.0 % [7 of 14]; CM, 35.5 % [12 of 34]) (Fig.****3****). A similar trend was seen for all 131 attacks. A greater proportion of HFEM attacks achieved*pain relief*at 1 h (HFEM, 45.5 % [15 of 33]; CM, 35.7 % [35 of 98]) and 2 h (HFEM, 60.6 % [20 of 33]; CM, 48.0 % [47of 98]); more attacks achieved*pain-free status*at 1 h (HFEM, 30.3 % [10 of 33]; CM, 13.3 % [13 of 98]) and at 2 h (HFEM, 33.3 % [11 of 33]; CM, 19.4 % [19 of 98]) (Fig.****4****).**

**The proportion of patients who responded to nVNS in ≥ 50 % of the migraine attacks at 2 h was 62.5 % for pain relief (78.6 % in HFEM, 55.9 % in CM) and 33.3 % for pain free (50 % in HFEM, 26.5 % in CM).**

At 2 h, freedom from nausea was reported in 66.4 % (87 of 131) of attacks; freedom from photophobia and phonophobia was reported in 76.3 % (100 of 131) and 77.1 % (101 of 131) of attacks, respectively. Complete recovery from functional disability at 2 h was reported in 35.1 % of attacks. Rescue medications were taken in 53.4 % (70 of 131) of the attacks.

No major adverse events were reported. Mild tingling or pricking sensations at the stimulation site, reported by 67 % (32 of 48) of patients, was the only adverse event associated with nVNS. Nearly half of the patients (45.8 %; 22 of 48) reported satisfaction (ie, *satisfied* or *very satisfied*) with treatment and were willing to use the device in the future. All patients considered nVNS treatment to be safe.

## Discussion

Results from the present study validate prior evidence that shows nVNS is effective for the acute treatment of migraine attacks in patients with HFEM or CM [11]. With enrollment of a larger (N = 50), more severely affected population who experienced more migraine attacks (N = 131), our research extends data from previous studies that showed a 2-h pain-free response of 22 % [11]. More than half of the patients (64.6 %) in our study experienced *pain relief* at 2 h, and 39.6 % were *pain free* at 2 h; a novel finding is the response to nVNS at 1 h, with 56.3 % of patients experiencing *pain relief*, including 35.4 % of patients who were pain free. **Additionally, we discovered that patients with a lower frequency of attacks (ie, HFEM; 8-14 headache days per month) appeared to achieve a better response than those with a higher frequency of attacks (CM; ≥15 headache days per month).** This finding represents an early treatment paradigm in which nVNS was administered when migraine pain was mild or moderate rather than severe. Although this paradigm may increase the placebo effect, it was selected because headaches in CM are typically reported to be mild or moderate compared with more severe headaches in episodic migraine [14, 15]. Moreover, persistent activity of pain-processing regions within the brain and low expectation of success in patients with CM may mitigate any placebo effect [16]. Other limitations of this study are the open-label design, lack of control group, and short duration. **Moreover, larger studies are required.** However, studies of nVNS in migraine [11] and cluster headache [17] have also implemented a short-term, single-arm, open-label design to demonstrate the feasibility of nVNS in real-world clinical practice. Preliminary data from large-scale, multicenter, randomized, controlled studies of nVNS in CM [18] and chronic cluster headache [19] have further corroborated its clinical benefit.

We investigated the benefit of nVNS in a real-world clinical setting; findings from this study will expand the body of clinical evidence on nVNS to the HFEM/CM population whose pain is difficult to manage. Furthermore, we implemented intensive educational training to ensure treatment adherence, assessed headache response at a short interval (ie, 1 h), and evaluated treatment satisfaction. Our data confirm that nVNS is well tolerated and safe and is associated with treatment satisfaction and therapeutic adherence. From a risk-benefit perspective, nVNS therapy achieved pain relief without serious side effects, which may decrease patients’ reliance on migraine medications and, in turn, lower the risk of medication overuse.
